# Three-Dimensional Modeling and *In Silico* Kinematic Evaluation of Interspinous Ligament Desmotomy in Horses

**DOI:** 10.3389/fbioe.2022.817300

**Published:** 2022-04-01

**Authors:** Adam Henry Biedrzycki, George Louis Elane

**Affiliations:** University of Florida, Gainesville, United States

**Keywords:** horse, spine, overriding, interspinous, desmotomy, kinematics, *in silico*

## Abstract

**Background:** Interspinous ligament desmotomy (ISLD) has been shown to improve the comfort of horses diagnosed with overriding dorsal spinous processes (DSP), but its effects on spine mobility are unknown.

**Objective:** To objectively quantify the change in mobility of thoracic vertebrae following ISLD using CT and medical modeling software.

**Study design:** Prospective cadaveric manipulation of seven equine thoracolumbar spines collected from T11-L1.

**Methods:** Spines were collected from T11-L1 with the musculature intact. Flexion and extension phases were achieved with a ratchet device calibrated to 2000N. Bone volume CT scans were performed in resting, flexion, and extension phase preoperatively. Interspinous ligament desmotomy was performed at each intervertebral space (*n* = 8), and bone volume CT imaging was repeated for each phase. The spinal sections were individually segmented and imported into medical software for kinematic evaluation. T11 of each phase were superimposed, the distance between each dorsal spinous process, the total length of the spine, and the maximal excursion of the first lumbar vertebra along with angular rotational information were recorded.

**Results:** The mean distance between each dorsal spinous process increased by 5.6 ± 4.9 mm, representing a 24 ± 21% increase in mobility following ISLD. L1 dorsoventral excursion increased by 15.3 ± 11.9 mm, craniocaudal motion increased by 6.9 ± 6.5 mm representing a 47 ± 36.5% and 14.5 ± 13.7% increase, respectively. The rotation of L1 about the mediolateral axis increased by 6.5^°^ post-ISLD.

**Conclusion and Clinical Relevance:** ISLD increases dorsoventral, craniocaudal, and rotational motion of the equine spine. The computer modeling methodology used here could be used to evaluate multiplanar spinal kinematics between treatments.

## Introduction

Impingement or overriding of the equine dorsal spinous processes (ORDSP), otherwise known as “kissing spines”, represents the most common source of thoracolumbar and back pain in horses (Jeffcott, 1980). Thoracolumbar vertebrae T10-18 are the most commonly affected, though the cranial lumbar vertebrae can occasionally be affected (Denoix and Dyson, 2011; Zimmerman et al., 2012). Presenting complaints of horses affected by back pain typically include a history of poor performance, pain on thoracolumbar palpation, and epaxial muscle atrophy in advanced cases. The assessment of pain on palpation of back musculature is subjective due to individual interpretation and techniques; pressure algometry is a more objective determination (Trager et al., 2020). Furthermore, the role and importance of musculature in horses with back pain are further highlighted when considering that postural changes to limit the dorsoventral range of motion (ROM) of the spine can occur by horses’ contraction of the epaxial muscles (Trager et al., 2020). Diagnosis of ORDSP can be challenging due to the low correlation of diagnostic imaging with clinical disease but is frequently characterized by an increase in radiopacity of the dorsal spinous processes (DSPs) on radiographs (Erichsen et al., 2004; Zimmerman et al., 2012; Trager et al., 2020). Diagnostic information can also be complemented by observing increased radiopharmaceutical uptake on nuclear scintigraphy (Erichsen et al., 2003). Additional diagnostics via ultrasonographic identification of fiber disruption of the ISL in horses with ORDSP is supported by a histological evaluation that reported loss of ISL integrity and fiber disruption in horses with ORDSP, although clinical correlation is not always apparent (Ehrle et al., 2019). Initial medical management typically consists of corticosteroid injection, muscle relaxants, and other analgesics, aimed at improving the comfort during the physical rehabilitation period. The available surgical treatments include surgical resection of the DSPs under general anesthesia, and subtotal ostectomy in the standing horse; however, these can result in poor cosmetic outcome and postoperative complications (Roberts, 1968; Perkins et al., 2005; de Souza et al., 2021). The strong supraspinous ligament runs along the length of the spinal column and must be elevated from the DSPs along with the mutifidis dorsi, spinalis thoracic, and longissimus dorsi muscles in order to permit the removal of a portion of the DSP (Rubio‐Martinez et al., 2021). The ISL occupies the space between the DSPs and interspinous ligament desmotomy (ISLD) has been shown to ameliorate back pain (Coomer et al., 2012) but its effects on increasing mobility of the spine are unknown. Several studies have shown a clinical improvement in horses suffering from poor performance post-ISLD (Prisk and García‐López, 2019; Brown et al., 2020), however, others have mentioned long-term complications such as unilateral neurogenic atrophy of epaxial musculature (Derham et al., 2021). The main effects of ISLD appear to be due to reduced tension on the afferent nociceptive receptors located at the ligament insertion, which abolish the pain response (Coomer et al., 2012). An increase in nerve fibers within the ISL itself has also been identified histologically, transection of which may contribute to a change in pain level (Ehrle et al., 2019). Radiographically, an enlargement of the interspinous space was observed after ISLD, although this was not quantified (Coomer et al., 2012). Furthermore, we do not know if the effect of ISLD has beneficial mobility changes or deleterious secondary biomechanical consequences; further studies are required to address these effects. Few studies have objectively evaluated the kinematics of the equine spine at the individual vertebral level, although more generalized kinematic studies have been performed (Townsend et al., 1983) and more recently kinematics between straight-line motion and circle in trot and with a rider have been evaluated (Martin et al., 2017; Byström et al., 2021). These studies utilized motion capture cameras and skin markers, which have certain limitations when applied (Pourcelot et al., 1998). This study aimed to quantify the postoperative change in the mobility of thoracic vertebrae using a combination of CT and medical modeling software using postmortem spine specimens from T11 to L1. The objectives were to explore and quantify post-ISLD changes in mobility in the following ways: 1) investigate DSP distance and length of spine sections, 2) investigate the mobility of each vertebral segment, and 3) investigate the vertical (dorsoventral) excursion of the first lumbar vertebrae. We hypothesized that 1) the distance between each DSP and overall length of the spine would be increased following ISLD, thereby signifying an increase in length or mobility in a craniocaudal distance and 2) the mobility between each DSP would increase post-ISLD and 3) the vertical excursion of the first lumbar vertebra (relative to T11) would be increased postoperatively, signifying an increase in mobility in the dorsoventral direction post-ISLD.

## Materials and Methods

### Study Design

**FIGURE 1:**
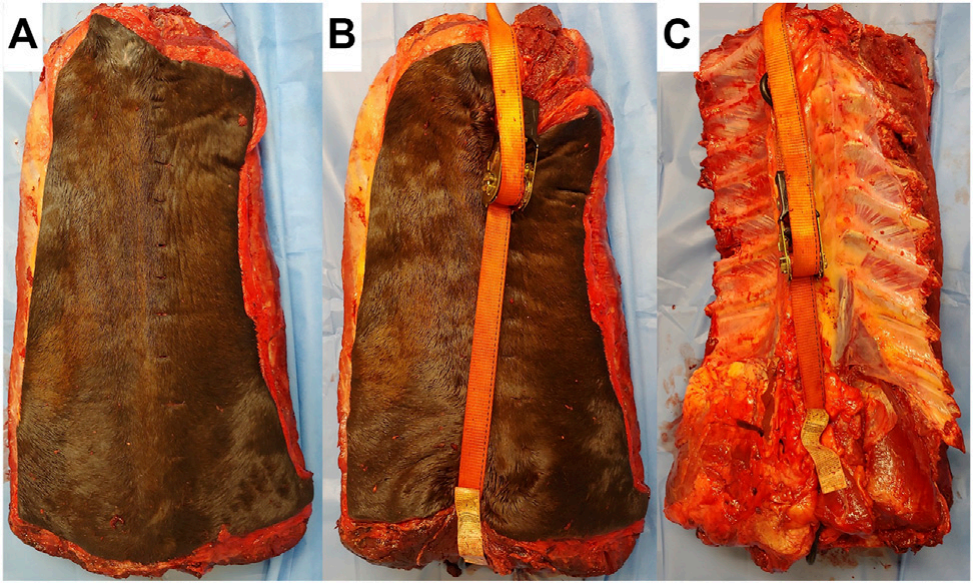
Images of spine sections prior to CT scanning. **(A)** Resting phase. **(B)** Flexion, ratchet strap calibrated to 2000N is placed on the dorsal aspect, compressing the dorsal spinous processes together. **(C)** Extension, ratchet strap is placed in the ventral aspect of the section, separating the dorsal spinous processes, and placing the interspinous ligament under tension.

**FIGURE 2:**
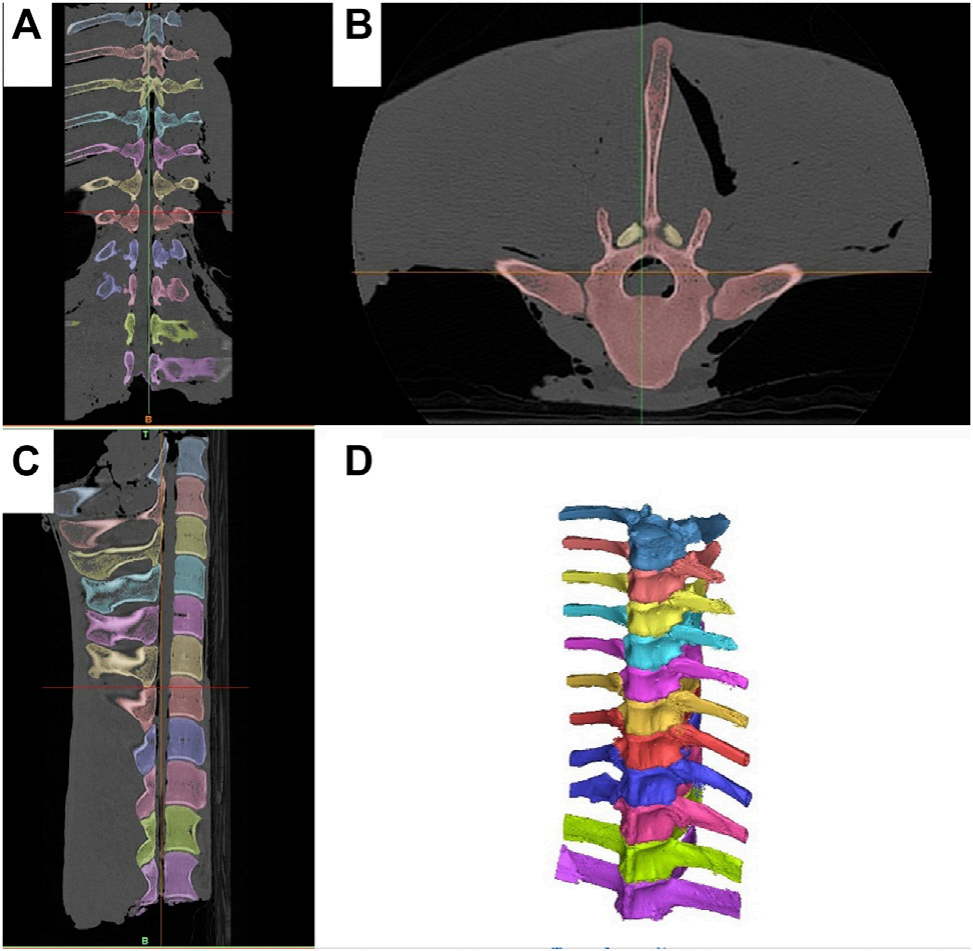
Segmentation of the T11-L1 spine segments. Each vertebra is segmented separately and denoted in a different color. **(A)** Dorsal CT scan view. **(B)** Axial CT scan view, **(C)** Sagittal CT scan view. **(D)** 3D reconstruction of each individual spinous process.

**FIGURE 3:**
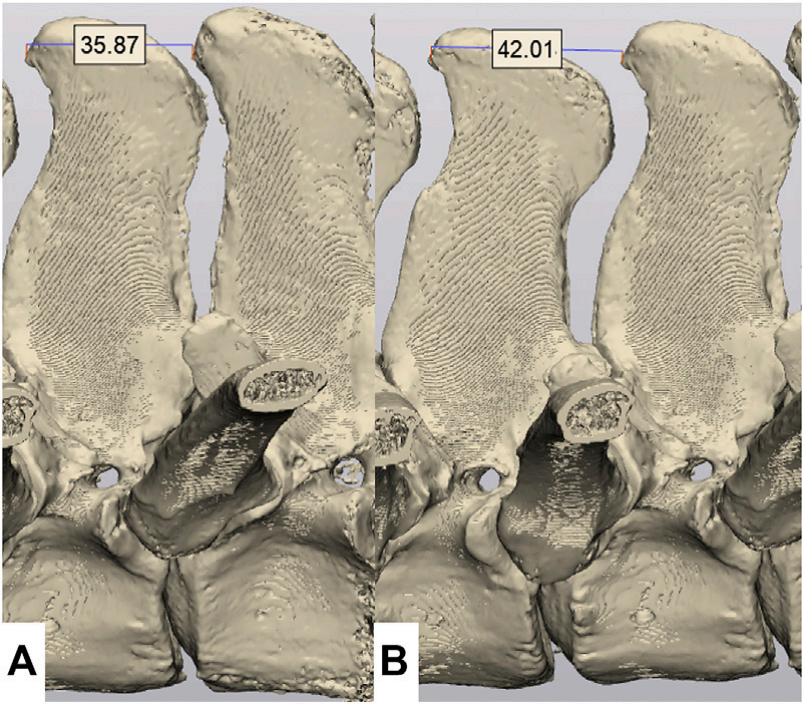
Measurement of the inter DSP distance. Measurements are made from the cranial aspect of one DSP to the cranial aspect of the adjacent DSP. **(A)** Flexion phase, demonstrating an inter DSP distance of 35.87 mm. **(B)** Resting phase, demonstrating a inter DSP distance of 42.01 mm.

**FIGURE 4:**
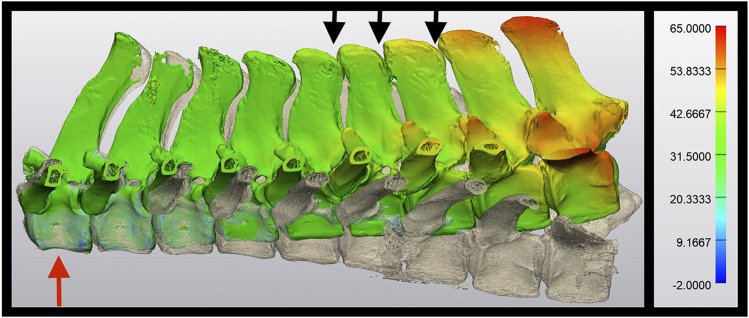
Use of Hausdorff distance to demonstrate dorsoventral excursion. In this image, T11vertebra (red arrow) have been superimposed over each other. The spine curving toward the top of the image represents a spine in extension, and the spine curving toward the bottom of the image represents the same spine in flexion. The color overlay represents the translation in mm between the two phases, peaking at 65.0 mm in this post-ISLD section. Note the close proximity of each DSP and narrowing of the interspinous space (black arrows) during the flexion phase.

Spines of seven skeletally mature horses with a body mass between 400 and 550 kg euthanatized for reasons unrelated to lameness or back pain were collected *en bloc* from T11-L1 with the skin, ventral and epaxial, and hypaxial musculature intact and the ribs severed at least eight inches from the spinal column (Figure 1). The spines were imaged and surgery was performed within 4 h of euthanasia, prior to the onset of rigor mortis. For CT evaluation (160 Slice Toshiba Aquillion CT Scanner, Cannon Medical Systems, Tustin, CA, United States) helical volume data (slice thickness of 0.5 and 0.3 mm slice overlap) was acquired. The bone reconstruction algorithm was used for all CT-based 3D reconstructions and analyses. The images were acquired in resting, extension, and flexion phases using a ratchet device before and after ISLD (Figure 1). The ratchet device was inserted into the spinal canal of the T11 vertebrae and the L1 Vertebrae and placed either on the dorsal or ventral aspect. The ratchet had a maximum strength of 2000N, verified with an inline force transducer and thus, the spine was loaded in each direction with 2000N. Based on CT imaging, the spines were screened for any preexisting ORDSP, inter DSP narrowing, or increased radio-opacity on the cranial and caudal margins of the DSPs and excluded if CT signs of these conditions were evident. ISLD was performed as previously described (Coomer et al., 2012). Briefly, 1 3.5” spinal needle was used to identify the interspinous space between each vertebra. Then, a 1 cm paramedian skin incision was made approximately 3 cm off midline using a #10 blade. Curved 7” Mayo scissors were inserted to bluntly penetrate the interspinous space and passed axially to transect the ligament, taking care not to damage the supraspinous ligament. This was repeated at each interspinous space n=8. The CT images in each position, both pre and post-ISLD were exported as DICOM files. The imaging data in DICOM files were imported into Materialise Mimics™ and 3-Matic™ (Materialise Medical Imaging Software Suite, Materialise NV, Leuven, Belgium) computer software for segmentation and 3D modeling of anatomic structures. In the software, each spinous process was individually segmented; the ribs were excluded (Figure 2). The initial process consisted of creating renderings, referred to as masks, using the Hounsfield Unit (HU) density range; for the spinous processes, an HU range of +700 to +3000 was used. Following the creation of the masks, 3D parts of each individual spinous section were created. These 3D renderings were then checked for imperfections and corrected with wrap and smooth algorithms. The parts were then imported into the spatial manipulation software program and the 11th thoracic vertebra of each phase was superimposed, maintaining the spatial relationship between them and each successive vertebra. Based on this data, the individual distance between the cranial-most aspect of each DSP (denoted by the most craniodorsal point), and the total spine length was measured and recorded in mm for each of the three phases (extension, flexion, and resting) at both the pre and post-ISLD time points (Figure 3). This provided information about the individual spinal motion. Finally, to gain an understanding regarding the global motion of the spine segments (T11-L1), the L1 in the extension and flexion phases were then superimposed over L1 in the resting position. This was achieved via a global registration algorithm, which aligns the features of different phase T11 sections within each horse resulting in a near-perfect alignment. The remainder of the spinal column segments within each phase move concurrently with respect to the parent T11 vertebrae, thus preserving the alignments. Following this, the translation and angular rotation of each phase (resting, flexion, and extension) was exported as a transformation matrix and Hausdorff distances of L1, the furthest vertebrae away from T11, were measured to demonstrate maximal changes between different phases (Figure 4) (Biedrzycki et al., 2021).

### Statistical Analyses

A test for normality was performed using Shapiro-Wilk. Data evaluating the interspinous distances were evaluated using a paired samples *t*-test. To determine the effects between pre and post-ISLD on each individual vertebra, a repeated measure 2-way analysis of variance with spinal section number and surgical condition (pre or post) as factors with a post-hoc Tukey’s test. Data were reported as mean ± standard deviation. Where data were not normally distributed, a Wilcoxon signed-rank test was performed, and data were reported as median (range). The significance was set at *p* ≤ 0.05. Statistics were performed with MedCalc software (Ver 19.1) and post-hoc power analysis was performed using G*Power.

## Results

### Horses

The spines from four Quarter horses, and one Thoroughbred, Warmblood, and Spotted Saddle Horse each were included in the study. The sexes comprised of four mares and three geldings with an age of 16.3 ± 3.3 years and weight of 461 ± 46.6 kg. All spines were included in the study and were considered normal; none met the criteria for exclusion.

### Resting Phase

There was no significant difference between the interspinous distance in the resting phase between each vertebra in the pre-surgical spine (39.89 ± 2.84 mm) and the post-surgical (39.90 ± 2.84 mm, *p* = 0.98). However, the power of this test was only 5%. To have sufficient power (80%) to detect a difference among the spinous process at rest given our effect size (0.003), a sample size approaching 2,000,000 sections would be required. Although 2,000,000 + sections are required to have sufficient power at a 20-micron difference, our study was sufficiently powered at 80% to determine that the true difference between the resting phases of the specimens we examined is less than 2.2 mm. Furthermore, we also identified a significant effect of vertebral space in our model (*p* < 0.01), indicating that the inter DSP distance is not homogenous among the vertebrae. However, even though we were unable to detect a difference at the interspinous level, there was a significant difference at the global level (T11-L1 range). After ISLD, the total distance between the cranial aspects of T11 to L1 is significantly increased (*p* = 0.03) from 310.0 ± 13.9 mm pre-surgery to 313.9 ± 11.8 post-surgery. The effect of ISLD on the total spinal length from T11 to L1 increases the length by 3.8 ± 3.1 mm. This 3.8 mm increase represents a 1.2 ± 1.0% change in the total length of the spine.

### Range of Motion—Individual Spine Segments

There was no significant difference in the inter DSP distance measured from the cranial aspect of one vertebra to the cranial aspect of the next vertebrae during extension between pre-surgical spines (27.4 ± 19.1 mm) and the post-ISLD spines (33.9 ± 9.0 mm, *p* = 0.32, Figure 3). However, for flexion, a significant increase in the inter DSP distance between pre-surgical (34.3 ± 17.4 mm) and the post-ISLD (44.4 ± 10.6 mm, *p* = 0.046) was identified. Combining these extension and flexion values to generate a total dorsoventral range of motion (ROM) for each individual space, we demonstrate that preoperatively, this ROM is 17.9 ± 3.0 mm. The effect of ISLD is to significantly increase this ROM to 23.5 ± 4.0 mm (*p* = 0.02). Thus the effect of ISLD at a single vertebral site was to increase the ROM between two adjacent vertebrae by 5.6 ± 4.9 mm. This distance represents a 24 ± 21% increase in maximal ROM post-ISLD.

### Global Spine Movement

L1 has significantly more total translation (excursion) in the dorsoventral direction between the pre-surgical (32.6 ± 10.8 mm) and the post-ISLD spine (48.0 ± 14.0 mm, *p* = 0.01). This represents an increase of 15.3 ± 11.9 mm of travel or an increase of 47.0 ± 36.5% in dorsoventral excursion. Extrapolating this figure, which represents the effects of 8 ISLDs, to a single ISLD at a single site provides an estimated effect of approximately 5.9% increase in dorsoventral excursion per ISLD site. L1 also has significantly more translation in the craniocaudal direction between the pre-surgical (22.9 ± 10.8 mm) and the post-ISLD spine (29.8 ± 10.9 mm, *p* = 0.03). This represents an increase of 6.9 ± 6.5 mm of travel or an increase of 30.1 ± 28.4% in craniocaudal translation. Extrapolating this figure to a single ISLD at a single site provides an estimated effect of approximately 3.8% increase in craniocaudal excursion per ISLD site. The angle of rotation of L1 between flexion and extension significantly increases from 12.0° (7.9–35.6°) pre-surgery to 21.8° (14.7–55.6°) degrees post-ISLD (*p* = 0.01). This represents an increase in the angular rotation of L1 of 6.5° (2.1–20.8°).

## Discussion

We successfully achieved our objectives, and this study represents the first *in silico* quantification of the change in mobility of thoracic vertebrae following ISLD using CT and medical modeling software. The lack of significant difference between individual spines in the preoperative and postoperative “resting” phases in this study suggests that this formed an adequate baseline for comparison of the flexion and extension phases. Despite this, we identified that there are differences in the interspinous distances between different vertebrae, although we did not have sufficient power to further determine where these differences exist during flexion and extension in this study. Furthermore, we also identified that the resting total length of the spine section we were studying (T11-L1) significantly increased by 3.8 mm, supporting our first hypothesis. However, this increase is approximately 1.2% of the total length of the spinal section analyzed and could be explained by thermal variations in the muscles and soft tissues over time and the initial flexion and extension phases, since the pre-surgical imaging was always performed prior to the surgical intervention.

The caudal thoracic and the lumbar spine is the least mobile region of the equine back (Townsend et al., 1983). However, this is the region at most risk for kissing spines and where surgical intervention is required. During the study, the determination of which specific desmotomy sites would be the most efficacious in increasing the overall mobility was considered. Although increasing mobility is not necessarily the clinical goal of the ISLD procedure, for the purposes of this study into the investigation of mobility we wanted to choose sites which would have most impact. However, a power calculation indicated that 1,309 spines would be required in order to make this determination, and the consideration was abandoned. Thus, in order to achieve a maximum global effect given the previously identified lack of mobility in this region, it was elected to perform ISLD at each intervertebral site (for a total of eight sites) in the current study, as opposed to the original description of the procedure, which had a mean of five surgical sites (Coomer et al., 2012). This approach was chosen in order to determine the maximum change in vertebral excursion following ISLD by increasing the number of desmotomy sites. Furthermore, we chose to measure our inter DSP distance from the cranial aspect of one vertebra to the cranial aspect of the next vertebrae rather than the distance between each DSP. This was performed due to issues selecting the optimal caudal marker, which was not reliably determined and varied based on the different computer views. To remove excessive variation, we elected to use only one point per spine, the most cranial and repeatable prominence on each DSP.

We identified that increased motion between the spinous processes occurs in the flexion phase in comparison to the extension phase during spinal motion in normal spines. Furthermore, there was no significant effect of ISLD on increasing the amount of motion in the extension phase; ISLD only had a significant effect on increasing the degree of motion in flexion. A limit of 2000N was used as a maximum value due to DSP fracture identified in preliminary studies if forces greater than 2000N were used in the extension phase. Although it is conceivable that forces greater than 2000N could have been applied in flexion, the authors decided to keep the forces consistent for direct comparisons between the two modes. This appears to make sense since, with transection of the ligament, one would not expect an increased motion when spines are compressed together (extension) but rather when they are placed in tension and forced apart (flexion). Thus, we found evidence to support our second hypothesis that the range of motion between each DSP would increase post-ISLD. Since our results demonstrate the effects on normal spine segments, horses with kissing spines exhibiting close proximity (similar to a type of “extension” phase) are likely to have greater effects of ISLD on mobility in the flexion direction than presented here, thus our results are likely to underestimate the clinical scenario. The constriction of the interspinous ligament on the mobility of the DSPs was first recognized by Coomer et al. who developed the ISLD technique (Coomer et al., 2012). Following ISLD, there was an increase in interspinous space observed on postoperative radiographs. They theorized that tension of the interspinous ligament alone was holding the DSPs in close proximity to each other, although the quantification of this change was not determined at that time. This ancillary finding of increased mobility is further supported by our study.

In terms of global spinal motion between T11-L1, we found evidence to support our third hypothesis. It stands to reason that the increase in dorsoventral excursion of the first lumbar vertebra by 15.3 mm following ISLD likely represents a cumulative increase in range of motion by severing the interspinous ligament between each preceding thoracic vertebra, thereby allowing the lumbar vertebra more freedom to move further in the dorsoventral direction. Similarly, the 6.9 mm increase in craniocaudal motion was statistically significant following ISLD and suggests that the interspinous ligament may constrict craniocaudal motion in addition to dorsoventral motion (Coomer et al., 2012). As demonstrated by this study, severing this ligament increased rotation of the lumbar vertebra by 6.5° supporting the notion that the ISLD increases the ability of the affected vertebrae to rotate further increasing overall spine mobility.

Histopathology of the interspinous ligament of horses affected by ISLD had revealed disruption of normal anatomy and fibrocartilaginous metaplasia in one report (Ehrle et al., 2019). The upregulation of fibrocartilage in affected horses may indicate a decrease in mobility, which predisposes to enthesiophyte formation and the osseous radiographic changes typically seen in horses with ORDSP. Furthermore, the number of nerves within the interspinous ligament was increased in horses with ORDSP in the same report (Ehrle et al., 2019). It is likely that nociception may play a part in decreasing mobility of the spine, and that severing these nerves consequently increases analgesia and allows the horse to engage in rehabilitative physiotherapy following ISLD.

The effect of ISLD is to enhance the mobility and rotation of the spine by approximately 24% per spinous segment in terms of total, maximal ROM in flexion and extension. Furthermore, the maximal excursion of the spine in a dorsoventral direction can increase by 5.9% at each ISLD site. It should be noted that these are maximal or extreme ranges of motion; at rest or physiological conditions, these changes represent a difference of only 1.6% in total spinal length and underscore the necessity of appropriate rehabilitation therapy in clinical cases (Prisk and García‐López, 2019). The original description of the ISLD technique emphasized the need for postoperative physical training to encourage epaxial muscle strength and core stability. The core strengthening exercises, after resolution of back pain, may increase the cross-sectional area of the spinal stabilizing musculature. If the spine is hypermobile post-ISLD as our study suggests, then a post-rehabilitation increase in support or spinal stabilization may be beneficial.

This study has several limitations. Many of our results have large SD values, which are only slightly smaller than the mean value itself. For example, the effect of ISLD on the total spinal length from T11 to L1 increases the length by 3.8 ± 3.1 mm. This 3.8 mm increase represents a 1.2 ± 1.0% change in the total length of the spine. However, the data are still significantly different, although there is a less than 3% chance of type I error. Although the data are significant and we have evidence to support our hypotheses, the authors have concern that there are systemic type I errors with our data; that the null hypothesis has been incorrectly rejected. This level of caution should be applied to interpretation of all of our data.

While the spine segments collected included the most commonly affected sites for ORDSP, these segments do not represent the entire vertebral column and therefore the inclusion of more vertebrae may change the results. Anchor points to the appendicular skeleton at the sacrum and in proximity to the scapula may also affect the results. Important muscles attachments at the ribs both cranially and caudally may also affect our results. Furthermore, there was no muscle tone in our postmortem specimens. It is believed that horses with ORDSP or back pain tend to have increased tone in the epaxial musculature and as such, drugs such as methocarbamol are often prescribed. This variation in muscle tone between our sections (no muscle tone), normal live horses (normal tone), and clinically affected horses (exaggerated muscle tone), may therefore have a significant role in the true motion of the spine in the clinically affected live horse. As such, we believe our results are maybe an overestimation of the true motion that occurs. In addition, our sections included the anticlinal vertebrae (T16) and thus there may be differences in extension and flexion from the vertebrae cranial and caudal to this maker, as spinous processes in humans with the S-shaped normal human spine have different sections undergoing extension and flexion concurrently. Furthermore, during these extension and flexion phases, the spinal segments undergo translation, axial rotation, and lateral flexion which, although not evaluated in the present study, could be easily investigated with the techniques utilized in this investigation. The specimens were collected from clinically normal horses, and translation and rotation results may change in horses clinically affected by ORDSP. In addition, the study was underpowered for detecting individual changes in vertebral motion, the ISLD procedure was performed at each interspinous site for 8 sites total and supraphysiological forces were exerted on the spine. This was to allow for global changes in mobility of the spine but may not directly correlate with changes seen in clinical practice, where typically fewer total sites of ISLD are performed and lower forces are exerted. The assumptions that global spine motion can be equally divided amongst individual vertebrae may be incorrect, as there are small alterations in the plane of motion of each facet joint of the spinal vertebrae. We feel that the contribution of facet joint alignment is likely to be minimal, however, given this uncertainty we urge caution in interpretation of these findings as they may not be true approximations of individual vertebral motion. In addition, changes in individual spine mobility are unlikely to be the primary reason for clinical improvement in horses after ISLD.

Despite these limitations, this is a powerful modeling technique that would allow for further evaluations of spinal motion in a virtual setting. One of the limiting factors currently is that it is not possible to CT the thoracolumbar spine of horses *in vivo* and furthermore, one would require dynamic flexion and extension positioning in the CT to acquire meaningful ROM data. Nevertheless, the information, once acquired, could be used in a variety of virtual modeling scenarios.

In conclusion, this study demonstrated that ISLD of 8 interspinous segments increases spinal mobility. It is uncertain if this change in mobility is beneficial or if there may be secondary biomechanical consequences, such as thoracolumbar facet disease, which are beyond the scope of this study. However, the techniques utilized in this study can be valuable for evaluating spinal kinematics in three dimensions both locally (individual vertebra) and globally (effects over the entire spinal segment).

## Data Availability

The raw data supporting the conclusion of this article will be made available by the authors without undue reservation.

## Abbreviations

CT, computed tomography; DICOM, digital imaging and communications in medicine; ISL, interspinous ligament; ISLD, interspinous ligament desmotomy; DSP, dorsal spinous process; ORDSP, overriding dorsal spinous processes; ROM, range of motion.
